# Identification of Children of Parents With Mental Illness: A Necessity to Provide Relevant Support

**DOI:** 10.3389/fpsyt.2018.00728

**Published:** 2019-01-08

**Authors:** Charlotte Reedtz, Camilla Lauritzen, Ylva V. Stover, Janita L. Freili, Kamilla Rognmo

**Affiliations:** ^1^Regional Centre for Child and Adolescent Mental Health and Child Wellfare, Faculty of Health Sciences, UiT The Arctic University of Norway, Tromsø, Norway; ^2^Institute of Psychology, Faculty of Health Sciences, UiT The Arctic University of Norway, Tromsø, Norway

**Keywords:** parents, mental illness, mental health care services for adults, risk factors, children

## Abstract

**Background:** The main objective of this study was to identify and describe core life circumstances of children with mentally ill parents (COPMI) and their parents. Knowledge about COPMI aged 0–17 years is necessary, as assessment of the risk and protective factors in their lives provide solid background for preventive interventions.

**Methods:** Participants (*N* = 422) were parents of minor children (*N* = 589) receiving treatment in the clinic for psychiatric illness and substance abuse at the University Hospital of Northern Norway. Data was drawn from electronic patient journals.

**Results:** A total of 286 mothers and 136 fathers participated in the study, and 46.3% were single parents. Parents had 1–7 children (*M* = 2.24; *SD* = 1.02). Most parents had one diagnosis (*n* = 311, 73.7%), and mood disorders was the most frequent type of diagnosis. The largest proportion of parents had serious mental disorders (*n* = 185; 46.0%), and a large proportion of the sample was affected by disorders of moderate severity (*n* = 156; 38.8%). The mean age of the children was 8.6 years (SD = 4.97), and 432 children (74.6%) had one or more siblings. The large majority of children had access to adult resource persons other than the mentally ill parent (*n* = 424; 94%), but 6% of the children (*n* = 27) did not. About three quarters of the children (76.2%, *n* = 526) were living with the mentally ill parent (*n* = 401), and 170 children (32.5%) lived with a single parent with a mental health disorder and siblings, full time or part of the time. The odds that parents had informed their children about the treatment/hospitalization and condition was higher the older the child was (*p* < 0.001), and the youngest children rarely got necessary information about this.

**Discussion:** Risk and protective factors associated with the children's ages, access to resource persons, information about the parent's health problems and treatment are discussed in relation to different preventive steps for COPMI.

## Introduction

Worldwide around onev in five minor children has a parent with a mental illness (1). In Norway it is estimated that 450,000 children have parents with a mental illness or substance use disorders (2). These children are at high risk of developing a mental illness themselves (3).

In a meta-analysis, (4) found that children of parents with a severe mental illness had a 50% chance of developing any mental illness, and 32% chance of developing a severe mental illness. In Norway, it has been estimated that children of parents with a mental illness (COPMI) have double the risk of both short-term and long-term negative consequences compared to children of parents without mental illnesses (2). Elevated risk has been documented for COPMI across the diagnostic spectrum of mental disorders in parents, including schizophrenia (5) obsessive-compulsive disorder (6), depression (7, 8), substance abuse disorders (9), anxiety disorders (10), bipolar disorder (11), eating disorders (12), personality disorders (13) and suicide (14). The transmission of risk for psychopathology from parents to children is both diagnosis-specific such that children may develop the same mental illness as their parents, and general, such that children are at risk of developing a wide range of disorders (10).

In addition to hereditary components of mental illness enhancing the risk of mental illness among the offspring, parents' symptomatology may also has a social impact and the ways parents interact with their children is therefore highly significant (8). Psychopathology in a parent often impairs parenting skills, the quality of care they provide and the parent-child interaction (15, 16). Such impairments may in turn lead to reduced involvement with the child, as well as insensitivity, hostility, rejection, neglect and potential abuse (17). The failure in one or more aspects of parenting can lead to insecure attachment (18, 19), emotional dysregulation, negative emotionality and pathological coping strategies (17), as well as psychopathology in childhood, adolescence and adulthood (20).

Different characteristics of the parent's psychopathology predict increased risk for the COPMI, including the symptom burden, comorbidity, and the severity and duration of illness. Empirical studies have repeatedly found that there is a greater negative impact on children whose parents have co-morbid disorders and personality disorders, compared to children whose parents have single disorders (21). Comorbid mental disorders in a parent may lead to larger and more long-lasting functional impairments, poorer prognosis and treatment complications 22, 23. Brennan et al. (24) found that children of depressed mothers were at greater risk of developing behavioral problems if the depression was severe, and that severity had a significant relationship to aspects of the children's language development. Although there are several risk factors related to parent's psychopathology, some COPMI are very resilient and are not impacted adversely. To our knowledge, no systematic reviews or meta-analyses have been conducted to quantify resiliency factors, but qualities of child personality and temperament, quality of attachment between the child and primary caregivers, as well as social support in the family and social network, is believed to buffer against adverse outcomes for COPMI.

It seems evident that the child's age is an important factor contributing to the outcomes for children at risk, and that younger children are at higher risk. For 1- and 2-years old children, depression in parents has been associated with impaired cognitive development, more behavioral problems, lower IQ scores in late childhood, as well as elevated rates of affective disorders in adolescence (8, 25).

The family is the core arena of development for children. However, the family situation for COPMI may be characterized by family conflicts, violence and negative life events (17, 26). Research findings have shown that children from conflict-ridden families were viewed less favorably by their peers and had fewer friends (26). In addition, COPMI often have care responsibilities in the household that exceed their emotional and cognitive maturity (27). Therefore, the risk for negative outcomes may be higher for children who live alone with one parent with mental illnesses, compared with those who live with both one parent with a mental illness and one healthy parent. Studies have found that single parents report having more mental health problems and behavioral problems compared with married parents (28). In contrast, the presence of a supportive and caring parent who understands the suffering of the ill parent can act as a buffer against negative child developmental outcomes related to depression in the mother (29).

Social and emotional ties to people outside the family can also moderate the effect of mental illness in parents (30), and the children's social network therefore plays a major protective role. Children benefit from having access to stable, non-familial trusted adults, such as teachers and other educational staff, as well as other adults in the child or parents' social support network and friends (31, 32).

Though impaired parenting as a result of parental psychopathology is a very potent risk factor for the development of emotional and behavioral problems in children, especially in early childhood, key parental functions are also modifiable (15, 16). Siegenthaler et al. (4) reported that family-focused interventions reduced children's risk of acquiring their own mental health problems by 40%. Another recent systematic review and meta-analysis reported small, but significant and lasting effects related to interventions for mothers and infants, as well as for children and adolescents themselves (20). Psycho-education is a common component across programs for COPMI and their families (33). The aim of such efforts is often to strengthen children's knowledge of the parent's psychopathology, as well as to reduce feelings of guilt and shame related to parental psychopathology. In general, research gives some indications that most parents do not speak with their children about mental illness. For example, a British survey showed that 55% of parents without mental illnesses and with children aged 6 to 18 did not talk with their children about mental illnesses.^1^ For COPMI, who are exposed to parental mental health symptoms on a daily basis, mental health literacy tend to be low (34), and they do not have access to accurate, non-stigmatized information about mental health disorders and treatments (35). Many COPMI do not seek help, neither from health care providers nor in their own network (36, 37). However, interviews of COPMI indicate that they want to be recognized as full-fledged members of the family by their parent's treatment providers, and to participate in the parent's therapeutic process to gain knowledge about the parent's illness and how to deal with it (36, 38). Many parents with mental illness are also concerned about the effects their mental illness may have on their children. They often want help, advice and guidance about how to talk to their children about their troubles.

In spite of this, COPMI receive little attention within mainstream mental health services (1, 34). In Norway, systematic routines for identifying these children did not exist until a few years ago (39). Based on knowledge about the transmission of mental disorders across generations, Norwegian authorities adopted amendments to the Health Personnel Act and Specialized Health Services Act in 2010. These provisions require health personnel in adult mental health services to identify and fulfill the needs of COPMI. It has taken time to implement these legal amendments, and they have thus far not led to satisfactory changes in clinical practice (40, 41). One study showed that 56% of health personnel at a large Norwegian university hospital did not identify patients' children (39), and a 5-years follow-up study showed that 28% of the health personnel in the same clinic still did not identify patients' minor children (42). Another recent Norwegian study showed that only 17% of patients in two psychiatric hospitals were assessed completely with family assessment forms (43). These results indicate that although the law requires identification of COPMI and provision of support in Norway, there has not been sufficient systematic work around implementing new clinical practice related to this issue in adult mental health care. Similar findings were reported in a study from adult mental health services in New Zealand (44).

The lack of identification of COPMI has large implications for public health as it is a core prerequisite to intervene in the high risk group of COPMI. Routine identification of COPMI in adult mental health services will provide necessary information about the children, their family situation and needs, and hence form the basis for provision of necessary family support and necessary professional collaboration across services and service levels in the municipalities where the family lives.

## The Norwegian COPMI Project—The Present Study

The COPMI project is a longitudinal research project in which the goal was to support the implementation of new routines arising from legislative amendments, as well as to evaluate the process of change (45). The project started in 2010, and involves a long-term strategy for changing clinical practice. The clinic initiated new procedures to identify COPMI by the use of *Family Assessment*. The *Family Assessment* form is an intervention for treatment providers to increase the identification of patients' minor children. The form consists of questions that the health personnel were required to collect as a result of legislative changes.

The main objective of the present study was to identify the children and basic life circumstances related to child development in families where the parent has a mental disorder, as well as to discuss how this information may be utilized to plan and strengthen professional collaboration in the provision of relevant support for COPMI.

These research questions were investigated:

(a) What are the demographic (gender, marital status, total number of children), and illness characteristics of the patients?
(b) What characterizes the children's gender, age, number of siblings, day care, and living arrangements, as well as the knowledge provided to them about parental mental illness and access to social supports?
(c) What is the relation between parent's diagnosis and disorder severity, and where the children live?
(d) Which factors influence whether or not the children get information about their parent's psychopathology?

## Methods

### Participants

The participants were 422 parents with mental disorders who received treatment at a clinic for psychiatric disorders and substance abuse at the University Hospital of North Norway (UNN). If the parent had more than one child, one assessment form per child was filled out. A total of 581 minor children were assessed.

### Data Material

The data consisted of information gathered by health personnel by using an adapted version of the information form “Family Assessment” (45). This is a standardized information form, designed to gather information about the child's gender, age, siblings, parental access, residence, and other caregivers who cared for the child during the parent's illness, as well as where the child was during the day and whether the child had received information about the parent's psychopathology. The form contained two questions about whether or not the children had been given information about the parent's treatment/hospitalization and the parent's condition. The response options for these questions were “yes,” “partially,” and “no.” In addition, ordinary electronic patient journals were assessed to gather information on the patient's gender, marital status, the total number of children, and diagnoses.

### Procedure

The information form was implemented as a compulsory routine for all staff in the participating departments and it was integrated in the electronic patient record. Under Norwegian law, collecting this information does not require consent.

The treatment provider filled in the information form in the electronic patient record during the conversation with the parent about the children. The treatment providers were psychologists, specialist psychologists, clinical social workers, clinical social educators, nurses, psychiatric nurses, activity therapists, doctors, psychiatrists, and social workers.

### Analysis

All analyses were performed using IBM SPSS Statistics 24. Descriptive frequency analyses were used to describe the sample. A chi-squared test was used to evaluate the relation between the parent's gender, whether the child lived alone with a parent with a mental illness or addiction, who cared for the child during any hospitalization, and whether the child had received information about the parent's illness. A chi-squared test was also used to look at the relationship between family composition and whether the child had other adult resource persons (i.e., the mentally healthy parent, a step parent, teachers, relatives, family friends, and/or neighbors).

Multivariate hierarchical logistic regression analyses were conducted to investigate the odds of having received information about the mental condition of the parent or about the parent being in treatment for children who lived with a single parent with a mental illness compared to other children. Multivariate hierarchical logistic regression analyses were also conducted to examine the relationships between information provided (about the mental condition and treatment), and the children's age, gender, and parental diagnosis. Interaction effects between the parental diagnosis and child age and gender were tested.

In order to compare whether the parent's diagnoses were connected to the various dependent variables (information about the disorder, receiving health care, living arrangements) in logistic regression analyses, the diagnosis variable was re-coded into dummy variables. Diagnoses that belonged to the same chapter in ICD-10 (46) were coded into the same dummy variables. Diagnostic categories with *n* < 15 were excluded in these analyses. This was true for F00–F09 Organic, including symptomatic, mental disorders (*n* = 5), F50–F59 Behavioral syndromes associated with physiological disturbances and physical factors (*n* = 5), F70–F79 Mental retardation (*n* = 1), F80–F89 Disorders of psychological development (*n* = 0), F99 Unspecified mental disorder (*n* = 0), F90–F98 Behavioral and emotional disorders with onset usually occurring in childhood and adolescence (*n* = 14), and R-/Z diagnoses (*n* = 11). In a regression analysis where the children's age was one of the variables, age was categorized in groups. In order to use the variables “has your child received information about your condition?” and “has your child received information about your health care/hospitalization?” as independent variables in a binary logistic regression, these were coded as dichotomous variables, where the response option “partially” was coded as “yes.”

The parents' primary diagnoses were categorized by their presumed severity. However, the distinction between serious and milder mental disorders is not firmly established in the discipline. The data set also did not contain information about daily functioning. The divisions used by Kessler et al. (22) was therefore adopted for this purpose, but without the functional goals. The diagnosis was coded as serious if it fulfilled one of the following criteria; assumed disability or significant limitations as a result of illness, drug addiction or if the disorder typically leads to 30 or more days per year in which the person is not able to maintain their roles socially, in the household, as an employee or as a partner. The diagnosis was coded as moderate if it did not meet any of the above criteria, but was considered to have a moderate impact on the aforementioned roles. All other disorders were classified as mild.

Logistic regression was used to examine the relationship between diagnoses and whether or not the child had received information about the parent's treatment and condition. To be able to compare the odds that the child had received information depending on the type of diagnosis, the diagnosis variable was recoded into dummy variables. Diagnoses that belonged to the same chapter in ICD-10 were coded in the same dummy variable, and diagnostic categories with *n* < 15 were excluded in these analyses.

## Results

### Characteristics of the Parents/Patients

#### Gender

The sample (*N* = 422) consisted of 286 mothers (67.7%) and 136 fathers (32.3%). The proportion of mothers was significantly larger than the proportion of fathers (*p* < 0.001).

#### Marital Status

The Family Assessment Form did not distinguish between being married or cohabiting. A total of 105 parents (43.4%,) reported to be married or living together with the other parent, 25 parents (10.3%) were married or living together with the child's step-parent, and 112 parents (46.3%) were single. Information about marital status was provided for 242 parents (57.3%), while for 180 parents (42.7%) information was missing.

#### Number of Children

Parents (*N* = 402) had between one and seven children (*M* = 2.24; *SD* = 1.02), whereas eight (1.9%) replied that they were expecting children.

#### The Parents' Diagnoses

The majority of parents had one diagnosis (*n* = 311, 73.7%), and some had two diagnoses (*n* = 70, 16.6%). A small proportion of parents had three diagnoses (*n* = 21; 5.0%), while few had no diagnosis (*n* = 20, 4.7%). The most commonly occurring diagnosis type were mood disorders (*n* = 190, 45%). Individually, the three most common diagnoses were F32.1 Moderate depressive episode (*n* = 60, 10.3%), F33.1 Recurrent depressive disorder, current episode moderate (*n* = 53, 9.1%) and F43.1 Post-traumatic stress disorder (*n* = 31, 5.3%).

#### Severity of Mental Illness

The largest proportion of parents in the sample had serious mental disorders (*n* = 185; 46.0%), followed by parents with disorders of moderate severity (*n* = 156; 38.8%). Mild mental disorders were the least common (*n* = 61; 15.2%).

### Characteristics of COPMI

#### Gender

The sample consisted of 290 (52.4%) boys and 263 (47.6%) girls. Information on gender was missing for 28 of the children (4.8%).

#### Age

The children's ages (*N* = 543) were evenly distributed from 0 to 17 years (Table 1). The average age was 8.6 years (SD = 4.97). A total of 76 children (14%) were below the age of 2 years old.

**Table 1:** Child age.

**Child age**	***N***	**%**
1–12 months	24	4.4
1 year	30	5.5
2 years	22	4.1
3 years	30	4.4
4 years	33	6.1
5 years	32	5.9
6 years	22	4.1
7 years	43	7.9
8 years	34	6.3
9 years	20	3.7
10 years	33	6.1
11 years	37	6.8
12 years	39	7.2
13 years	34	6.3
14 years	32	5.9
15 years	25	4.6
16 years	31	5.7
17 years	22	4.1
Total	543	100
Age missing	39	6.7

#### Siblings

A total of 432 children (74.6%) had one or more siblings (*M* = 1.25; *SD* = 1.04), and 382 children (66.0%) had one or more siblings under the age of 18, whereas 93 children (16.1%) had one or more siblings above the age of 18. A total of 147 children (25.4%) had no siblings.

#### Custody For the Child

A total of 186 children (38.9%) lived with both parents, whereas 83 children lived with each parent in a 50–50% manner. A total of 146 children (30.5%) lived with their mother, and 29 (6.1%) with their father.

#### Access to Adult Resource Persons

Most parents stated that their children had access to adult resource persons other than the mentally ill parent (*n* = 424, 94%). A total of 6% of the children (*n* = 27) did not have this. A chi-squared test was used to see if there was a relationship between being a single parent with a mental illness and whether or not the children had other adult resource persons. The results showed that there was no significant difference; children who lived with a single parent with a mental illness were just as likely to have other close adults as children in homes with two parents, step-parents or other adults.

#### Care For the Child When the Mother/Father Is in Treatment/Hospitalized

In sum, one or both the parents cared for the child (*n* = 308; 71.7%) when one of them received treatment/was hospitalized (Table 2). The majority of the remaining children were taken care of by other family members. If the father was in treatment/hospitalized, the child's mother cared for the child in 88.8% of the cases, whereas if the mother was hospitalized, the child's father cared for the child in 62.7% of the cases, which was a significant difference (χ^2^(2, N = 430) = 31.348, *p* < 0.01). Children with mentally ill mothers were more likely to be cared for by grandparents, stepparents and other family members (17.1%) than children with mentally ill fathers were (4.2%).

**Table 2:** Caregivers when mother/father is receiving treatment/hospitalized.

**Caregiver**	***N***	**%**
Mother	128	29.8
Father	180	41.9
Grandparent/s	24	5.6
Other family member/s	22	5.1
Fosterparent/s	20	4.7
Emergency placement	9	2.1
Stepparent	9	2.1
Friends/others	4	0.9
Child lives alone	3	0.7
Not applicable	31	7.2
Missing	149	25.6

#### Children Living With Mentally Ill Parent

About three quarters of the children in the sample (76.2%, *n* = 526) were living with the mentally ill parent. If the patient was the father, a bigger proportion of the children did not live with him (47.3%) than if the patient was the mother (12.6%).

#### Children Living With a Single Parent With a Mental Illness

A total of 170 of the children (32.5%) lived with a single parent with a mental illness and any siblings, either all the time (*n* = 91, 17.3%) or part of the time (*n* = 80, 15.2%). A greater percentage of the children with a mentally ill mother lived alone with her and any siblings, as compared to the children with a mentally ill father, and this difference was significant (χ^2^(2, N = 526) = 44.547, *p* < 0.001).

#### Children Living Outside the Home

A total of 45 children (7.8%) lived with neither their mother nor their father. The majority of these children either lived in a foster home (*n* = 21; 46.7%) or in an emergency placement home (*n* = 9; 20%). The other children (*n* = 15; 33.3%) lived with grandparents or siblings of the parents.

#### School and Day Care

Most of the children were at school (*n* = 304, 67.6%) or in kindergarten (*n* = 119, 26.4%) during the day. The others were at home (*n* = 23, 5.1%), in an emergency placement home (*n* = 3, 0.5%) or in a youth home (*n* = 1, 0.2%). Among the children who were at home during the day, 14 were under the age of 2 years.

## Parent's Diagnosis and Disorder Severity, and Its Relation to Where the Child Lives

### Parent's Diagnosis

Logistic regression showed that F10–F19 *Mental and behavioral disorders due to psychoactive substance use* was the only significant diagnostic category that could predict children's living conditions, and the odds that the child *did not* live with the parent was higher for parents with diagnoses in this category (*n* = 44; OR = 8.7; *p* < 0.001).

### Diagnosis Severity

Logistic regression also showed that the odds that the child did *not* live with the parent was 3.3 times higher for the most severe mental disorders (*p* < 0.001) compared to mild disorders.

## Factors That Affect Whether Children or Not Children Are Informed About the Parent's Treatment/Hospitalization and Condition

The parents of a total of 67.8% of children (*N* = 395) were asked “*Does your child know that you receive treatment/does your child know that you are hospitalized*?” Of these, a total of 54.9% (*n* = 217) responded “yes,” 6.8% (*n* = 27) answered “partially,” and 38.2% (*n* = 151) replied “no.”

The question “*Has your child received information about your condition?*” was answered by the parents of a total of 61.6% of the children (*N* = 359). Of these, a total of 44% (*n* = 158) responded “yes,” 14.5% (*n* = 52) answered “partially,” and 41.5% (*n* = 149) replied “no.”

### Family Composition

Children who lived with a single parent with a mental illness were more likely to receive information than the children living with both parents. The results from a hierarchical logistic regression showed that if the child lived with a single parent with a mental illness, the odds that the child had been given information about both treatment/hospitalization (Table 3) and the condition (Table 4) were higher. An analysis of whether there were interaction effects between the parent's gender and marital status in how much information the child received, showed no such interaction.

**Table 3:** Relation between information provided for the child about parent's treatment/hospitalization and whether the child lives with a single mentally ill parent or not.

	***X***^****2****^**(2)** **=** **12,225**, ***p*** **=** **<** **0.002**		
	**OR^a^ [95% CI^b^]**	**Wald**	***p***
Part time	1.93 [1.05, 3.56]	4.43	0.035^**^
Yes	2.55 [1.36, 4.78]	8.60	0.003^*^

a *Odds ratio*.

b *Confidence interval*.

* *Significant at 0.01 level*.

** *Significant at 0.05 level*.

**Table 4:** Relation between information provided for the child about parent's condition and whether the child lives with a single mentally ill parent or not.

	***X***^****2****^**(2)** **=** **12,663**, ***p*** **=** **<** **0.002**		
	**OR*^a^* [95% CI^b^]**	**Wald**	***p***
Part time	1.73 [0.92, 3.29]	2.88	0.090
Yes	2.87 [1.50, 5.50]	10.13	0.001^*^

a *Odds ratio*.

b *Confidence interval*.

* *Significant at 0.01 level*.

Logistic regression was used to examine the relationship between the information provided and the children's age. The odds that parents had informed their children about the treatment/hospitalization (Table 5) and condition (Table 6) was higher for each age group (*p* < 0.001); The odds of 15–17-years-olds having received information about the treatment/hospitalization was 43.77 times higher than for 0–2-years-olds, and the odds that they had received information about the condition was 47.78 times higher.

**Table 5:** Relation between information provided for the child about parent's treatment/hospitalization and child age.

	***X***^****2****^**(5)** **=** **96.128**, ***p*** **=** **<** **0.001**		
**Child age**	**OR^a^ [95% CI^b^]**	**Wald**	***p***
3–5	5.06 [1.97, 12.98]	11.37	0.001^*^
6–8	7.93 [3.14, 20.02]	19.19	0.000^*^
9–11	13.51 [5.19, 35.22]	28.39	0.000^*^
12–14	29.77 [10.98, 80.70]	44.48	0.000^*^
15–17	43.77 [14.73, 130.03]	46.27	0.000^*^

a *Odds ratio*.

b *Confidence interval*.

* *Significant at 0.01 level*.

**Table 6:** Relation between information provided for the child about parent's condition and child age.

	***X***^****2****^**(5)** **=** **95.446**, ***p*** **=** **<** **0.001**		
**Child age**	**OR^a^ [95% CI^b^]**	**Wald**	***p***
3–5	4.46 [1.51, 13.14]	7.34	0.007^**^
6–8	9.62 [3.37, 27.44]	17.92	0.000^*^
9–11	13.37 [4.56, 39.19]	22.34	0.000^*^
12–14	39.00 [12.75, 119.35]	41.22	0.000^*^
15–17	47.78 [14.48, 157.65]	40.29	0.000^*^

a *Odds ratio*.

b *Confidence interval*.

* *Significant at 0.01 level*.

** *Significant at 0.05 level*.

### Children's Gender

There were no significant differences between boys and girls in how much information they had received about the parent's treatment/hospitalization and condition.

### Parent's Gender

Results from a chi-squared test showed differences between children with mentally ill mothers and children with mentally ill fathers in terms of how much information they had received about the parental illness. There were significant differences based on the parent's gender in how much information the child had received about the treatment/hospitalization (χ^2^(2, N = 316) = 8.606, *p* < 0.05). There were also significant differences in whether or not they had received information about the parent's condition (χ^2^(2, N = 349) = 10.015, *p* < 0.01). For both variables, the children of mentally ill mothers were more likely to have received information compared to children of mentally ill fathers.

### Parent's Diagnosis

The odds ratio that the child received information about the parent *treatment/hospitalization* were highest if the parents had diagnoses from the categories F20–F29 Schizophrenia, schizotypal and delusional disorders (OR = 2.8; *p* > 0.05) (Table 7), and F60–F69 Disorders of adult personality and behavior (OR = 3.2; *p* = 0.08). For the diagnostic category F10–F19 Mental and behavioral disorders due to psychoactive substance use, the odds of information being given were nearly halved (OR = 0.5; *p* = 0.22).

**Table 7:** Relation between information provided for the child about parent's health condition and parent's diagnosis.

		***X***^****2****^**(5)** **=** **18,665**, ***p*** **=** 0.002^*^		
	***N***	**OR^a^ [95% CI^b^]**	**Wald**	***P***
F10–F19 Mental and behavioral disorders due to psychoactive substance use	44	2.56 [0.82, 7.97]	2.62	0.106
F20–F29 Schizophrenia, schizotypal and delusional disorders	59	8.31 [2.75, 25.09]	14.09	0.000
F30–F39 Mood [affective] disorders	268	3.70 [1.62, 8.44]	9.63	0.002
F40–F48 Neurotic, stress-related and somatoform disorders	121	4.62 [1.86, 11.45]	10.93	0.001
F60–F69 Disorders of adult personality and behavior	25	3.65 [1.06, 12.56]	4.22	0.040

a *Odds ratio*.

b *Confidence interval*.

* *Significant at 0.005 level*.

The odds ratio that the child received information about the parent's *condition* were highest when the diagnosis fell within F20–F29 Schizophrenia, schizotypal and delusional disorders (OR = 8.3; *p* = < 0.0005) (Table 8).

**Table 8:** Relation between information provided for the child about severity of parent's diagnose, child age, child gender and parent's treatment/hospitalization.

	**Model 1**			**Model 2**			**Model 3**			**Model 4**		
	***X***^****2****^**(2)** **=** **3.510**, ***p*** **=** **0.173**			***X***^****2****^**(4)** **=** **83.096**, ***p*** **<** **0.001**			***X***^****2****^**(6)** **=** **85.323**, ***p*** **<** **0.001**			***X***^****2****^**(8)** **=** **87.086**, ***p*** **<** **0.001**		
	**OR^a^ [95% CI^b^]**	**Wald**	***p***	**OR [95% CI]**	**Wald**	***p***	**OR [95% CI]**	**Wald**	***p***	**OR [95% CI]**	**Wald**	***p***
Severity: Mild	Reference group											
Severity: Moderate	1.40 [0.74, 2.65]	1.06	0.303	1.51 [0.740, 3.10]	1.28	0.257	0.48 [0.08, 2.72]	0.70	0.404	0.75 [0.10, 5.65]	0.08	0.781
Severity: Severe	1.81 [0.95, 3.45]	3.27	0.071	1.56 [0.85, 3.59]	2.29	0.130	0.99 [0.1, 5.50]	0.00	0.992	1.83 [0.25, 13.51]	0.35	0.553
Child age				1.92 [1.64, 2.25]	63.75	0.000	1.56 [1.05, 2.30]	4.94	0.026	1.60 [1.07, 2.39]	5.29	0.022
Child gender (1 = boy)				1.00 [0.62, 1.61]	0.00	0.998		0.00	0.967	1.93 [0.57, 6.52]	1.12	0.290
Mild*age								2.23	0.327		1.92	0.384
Moderate*age							1.41 [0.88, 2.26]	2.04	0.153	1.37 [0.85, 2.22]	1.66	0.198
Severe*age							1.18 [0.75, 1.85]	0.49	0.486	1.15 [0.72, 1.82]	0.33	0.567
Mild*gender											1.73	0.420
Moderate*gender										0.53 [0.13, 2.21]	0.76	0.385
Severe*gender										0.39 [0.09, 1.60]	1.72	0.190

a *Odds ratio*.

b *Confidence interval*.

### Diagnosis Severity

A multivariate hierarchical logistic regression was used to examine the relationship between the severity of the parent's diagnosis (mild, moderate, severe), the child's age and gender, and whether the child had been given information about the treatment/hospitalization (Table 9) and the parent's condition (Table 10). In the first model, the severity of the diagnosis was added alone, while in model 2 the child's gender and age were added. In the last model, the interaction effects were also examined. The interaction effects were examined in individual analyses. The odds that someone had informed their children about their treatment/hospitalization was greater the more serious the diagnosis was, but this difference was not significant. The odds that someone had informed their children about the parent's condition were also greater the more serious the diagnosis was, but this difference was not significant.

**Table 9:** Relation between information provided for the child about severity of parent's diagnose, child age, child gender and parent's health condition.

	**Model 1**			**Model 2**			**Model 3**			**Model 4**		
	***X***^****2****^**(2)** **=** **5.246**, ***p*** **=** **0.073**			***X***^****2****^**(4)** **=** **88.778**, ***p*** **<** **0.001**			***X***^****2****^**(6)** **=** **92.552**, ***p*** **<** **0.001**			***X***^****2****^**(8)** **=** **95.957**, ***p*** **<** **0.001**		
	**OR^a^ [95% CI^b^]**	**Wald**	***p***	**OR [95% CI]**	**Wald**	***p***	**OR [95% CI]**	**Wald**	***p***	**OR [95% CI]**	**Wald**	***p***
Severity: Mild	Reference group											
Severity: Moderate	1.50 [0.77, 2.91]	1.41	0.235	1.50 [0.70, 3.19]	1.08	0.300	0.29 [0.05, 1.75]	1.83	0.176	0.27 [0.04, 2.01]	1.63	0.202
Severity: Severe	2.12 [1.08, 4.15]	4.75	0.029	1.94 [0.90, 4.19]	2.88	0.090	0.47 [0.08, 2.83]	0.68	0.410	0.69 [0.10, 4.93]	0.14	0.712
Child age				2.05 [1.72, 2.45]	64.36	0.000	1.42 [0.96, 2.10]	3.01	0.083	1.42 [0.96, 2.11]	3.03	0.082
Child gender (1 = boy)				0.97 [0.59, 1.60]	0.02	0.904	0.96 [0.58, 1.60]	0.02	0.880	1.25 [0.37, 4.22]	0.13	0.714
Mild*age								4.06	0.131		4.18	0.123
Moderate*age							1.62 [1.00, 2.63]	3.79	0.052	1.62 [1.00, 2.64]	3.78	0.052
Severe*age							1.51 [0.93, 2.45]	2.82	0.093	1.55 [0.95, 2.52]	3.08	0.079
Mild*gender											0.05	0.188
Moderate*gender										1.19 [0.28, 5.02]	1.31	0.817
Severe*gender										0.43 [0.10, 1.84]	2.81	0.253

a *Odds ratio*.

b *Confidence interval*.

**Table 10:** Relation between information provided for the child about severity of parent's condition, child age, child gender.

	**Model 1**			**Model 2**			**Model 3**			**Model 4**		
	***X***^****2****^**(2)** **=** **5.246**, ***p*** **=** **0.073**			***X***^****2****^**(4)** **=** **88.778**, ***p*** **<** **0.001**			***X***^****2****^**(6)** **=** **92.552**, ***p*** **<** **0.001**			***X***^****2****^**(8)** **=** **95.957**, ***p*** **<** **0.001**		
	**OR^a^ [95% CI^b^]**	**Wald**	***p***	**OR [95% CI]**	**Wald**	***p***	**OR [95% CI]**	**Wald**	***p***	**OR [95% CI]**	**Wald**	***p***
Severity: Mild	Reference group											
Severity: Moderate	1.50 [0.77, 2.91]	1.41	0.235	1.50 [0.70, 3.19]	1.08	0.300	0.29 [0.05, 1.75]	1.83	0.176	0.27 [0.04, 2.01]	1.63	0.202
Severity: Severe	2.12 [1.08, 4.15]	4.75	0.029	1.94 [0.90, 4.19]	2.88	0.090	0.47 [0.08, 2.83]	0.68	0.410	0.69 [0.10, 4.93]	0.14	0.712
Child age				2.05 [1.72, 2.45]	64.36	0.000	1.42 [0.96, 2.10]	3.01	0.083	1.42 [0.96, 2.11]	3.03	0.082
Child gender (1 = boy)				0.97 [0.59, 1.60]	0.02	0.904	0.96 [0.58, 1.60]	0.02	0.880	1.25 [0.37, 4.22]	0.13	0.714
Mild*age								4.06	0.131		4.18	0.123
Moderate*age							1.62 [1.00, 2.63]	3.79	0.052	1.62 [1.00, 2.64]	3.78	0.052
Severe*age							1.51 [0.93, 2.45]	2.82	0.093	1.55 [0.95, 2.52]	3.08	0.079
Mild*gender											0.05	0.188
Moderate*gender										1.19 [0.28, 5.02]	1.31	0.817
Severe*gender										0.43 [0.10, 1.84]	2.81	0.253

a *Odds ratio*.

b *Confidence interval*.

Model 2 shows that the child's age could predict whether the child received information or not, which was also shown in the results of the *t*-test, but no interaction effect was found between diagnosis severity and age of the child (Model 3). The child's gender did not affect the information the child received, and there was no interaction effect between diagnosis severity and the child's gender when it came to whether or not the child had been given information about the parent's treatment/hospitalization or condition.

## Discussion

### Characteristics of the Patient/Parents

Parenting is challenging in itself, and a large number of studies indicate that parents with mental illnesses struggle more with parenting than parents who does not experience mental illness (16, 47). Nearly half of the patients in this study were single, and this may result in both a greater scope of problems in the parent (28), lower SES (48), and possibly an increased risk of the children developing mental disorders (49). In addition to their parent's mental illness, children who also have been through a divorce between their parents may have experienced a stressful and risk-enhancing transition in their family life, because of conflicts, moving and breaks in family relationships (50).

The largest proportion of the parents with mental illnesses were mothers, which is consistent with findings showing that more women than men seek help for mental health problems (51). Mood disorders, such as different forms of depression were most common in the sample, and the diagnosis F33 Major depressive disorder (recurrent), recurrent, was listed as the second most common illness. Recurrent and chronic disorders have been shown to lead to higher risks for COPMI (52, 53).

Most parents in our study had serious psychiatric disorders, which add to the high risk in these patients' children, as several studies have found a significant relationship between severity and chronicity of the parent's mental illness and the risk of negative outcomes in their children (3). In addition, mental disorders of moderate severity may lead to significant functional decline (2) and develop into more serious disorders over time.

The implications of the findings regarding the patients in this study is that the majority of patients are characterized by factors that predict high risk for their offspring. Therefore, health professionals in the clinic need to implement family support to reduce these risks and prevent developmental problems in the COPMI. Specifically, there is need for interventions which can be tailored to single parents with moderate to severe mood disorders, as well as recurrent disorders. The finding that parents with mental and behavioral disorders due to psychoactive substance use are less likely to have their children at home, also emphasize the need for interventions that suit these families' needs. It seems evident that these parents and their children need support to communicate and engage with each other in safe ways, as well as support if they are moving together again.

### The Characteristics of the Children and Their Living Circumstances

One third of the children in this study were 0–5 years old, one third were 7–11 years old and one third were 12–18 years old. Fourteen percent of them were 2 years or younger, and as such within the 1,001 critical days defined by the well-known manifesto “The Importance of the Conception to Age Two Period” from the UK.^2^ This manifesto, and others of its kind, highlights the importance of intervening early to enhance the outcomes for children at risk, and this is also a key objective for the development and implementation of interventions in Norway.

It seems fair to assume that most of the children under six in our study probably do not need outpatient help for their own psychological problems yet. However, unless they are identified and can access necessary support and preventive measures by health professionals treating the parent, only some of those who develop social and emotional problems themselves will be reached at a later point in life, and only after the problems have escalated to the point where the child needs to be referred to the specialist health service. This is demonstrated by the national figures for children who receive treatment in Child and adolescent mental health services (CAMHS), where < 7% of patients are boys under 7 years, and < 5% are girls below the age of seven. Less than 1% of patients in Norwegian CAMHS are under 4 years of age.

At the same time as the youngest children largely do not receive treatment by the mental health services for children and young people, they are especially vulnerable developmentally. Once problems have emerged, they have the worst prognosis and there are relatively few documented effective interventions and measures designed for this age group (4). In this study, no information was collected about how old the child was when the parent's illness was presented. However, recurrent depression was a common disorder in the sample, and for the youngest children, it is therefore reasonable to assume that a large part of their childhood had been affected by the parent's illness. In addition to this, a large majority of COPMI in the present study lived with the ill parent alone, either all the time or part time. As depression is a prevalent disorder in general psychiatric clinics, and has a documented negative effect on parent-child interaction (25), especially for the very youngest (17), feasible interventions for depressed parents of very young children should be a priority in general psychiatric clinics in Norway. Collaboration between health personnel in adult mental health services and public nurses in local health clinics, as well as preschool teachers in kindergartens seems especially valuable for this group of children.

Positive social bonds between siblings are an important source of protection for children who undergo stressful life events (54). The majority of our sample had one or more siblings. Sibling bonds can be considered especially beneficial for COPMI with siblings who are older than 18 (16.1% in our sample), where the sister or brother can function as a supporting adult (55). At the same time, dysfunctional sibling bonds, which are not uncommon in families with mental illness, may be a burden and can predict a subsequent psychopathology for COPMI (56). This suggests that sibling relationships between COPMI should also be assessed in mental health services for adults. In our sample, 7.5% of the parents had four or more children, and account must be taken of the negative impact that a larger number of children can have on the family economy, thus contributing to the socio-economic risk (57), as well as the increase in parental burden and need for relevant support for patients with more children. Labor and Welfare Services should be a natural collaboration partner for health personnel in adult mental health services in these regards.

The majority of the children had access to adult resource persons other than the parent with mental illness or addictions. It was usually the other parent or extended family who cared for the child when the parent with a mental illness was hospitalized. In addition, most of the children were at school or in kindergarten during the day. Only a small proportion of the children in the sample were at home during the day (5.1%). These findings emphasize how important the extended family, and personnel in kindergartens and schools are as significant others who may support the child's development. However, the provision of necessary information about the risks and protective factors relevant for each COPMI is crucial to motivate and engage the resources that significant others may contribute with. Child protection services (CPS) may intervene in those families where the children do not have access to resource persons beside the mentally ill parent. However, in a recent study we found that personnel in adult mental health services was reluctant to refer families of concern to the CPS (58). In Norway around 80% of the activity in public child protection services constitutes of preventive and compensating interventions. Examples of such interventions are economic support, practical support for parents and children in the home and related to leisure time activities, visitation homes, as well as parent training interventions offering supervision and guidance for parents in need of professional help to change their parenting practices. Barriers to inter-service collaboration based on lack of knowledge about the CPS in adult mental health services has serious consequences for families in need of support and for dissemination of interventions in the communities they live. Results from our research indicate that there is an unresolved potential for inter-service collaboration involving the children of patients with mental health problems. There is a large potential for improvement in the collaboration between adult mental health services and community services for children and their families in Norway.

### COPMI's Family Status and Factors Influencing Their Living Arrangements

Several factors were found that had an impact on the children's living arrangements and factors that led to an increased chance that the child did *not* live with the mentally ill parent were: (1) that the parent had serious mental illness, as compared with mild mental illness, (2) that the parent had an addictive disorder, and (3) that the ill parent was the father.

The severity of the diagnosis was a strong predictor and the odds were three times as high that a child did not live with a parent with a serious mental disorder compared to those with mild mental disorders. This is not a surprising finding, as the more serious mental disorders often lead to higher symptom pressure and malfunction, which to a greater extent leads to a risk of negative outcomes for the child. However, about three quarters of the children in our sample lived with the parent with a mental illness, and one third of them lived alone with this parent all the time or part time. These children will be more vulnerable and at a higher risk for developing mental disorders, as compared with those who also lived with a healthy parent (28).

The COPMI whose parents had addictive disorders were distinct from other COPMI, as their odds of not living with the ill parent were many times higher than for the children of parents whose primary diagnose were from the other categories. This was irrespective of the parent's gender. The reason why families and/or professional helpers choose to move children away from addictive parents is probably associated with substance abuse leading to risk in several different areas, in terms of a problematic social network, crime, physical and mental health problems, impulsive absences from the home, financial problems, on top of challenges with interactions when intoxicated.

About half of the children of mentally ill fathers lived with their fathers, while nearly nine out of ten children of mentally ill mothers lived with their mothers. The majority of children who lived with a single parent with a mental illness lived with their mother. In Norway, most children stay with their mother after a divorce between their parents (59), and it is therefore not possible to say whether it is the break-up or the father's illness that explains this. On the other hand, a previous study showed that mental disorders in fathers may reduce the involvement they have with their children (47). Taken together, our findings indicate that interventions supporting parents with severe mental disorders and their minor children, especially mothers, should be implemented. Service collaboration between community and specialist services is needed to target each family's needs in their everyday lives.

### Factors Influencing Whether or Not COPMI Learn About Their Parent's Illness

Half of the children in the clinic had received information about the parent's condition and treatment/hospitalization. Four of ten COPMI had *not* received information about the parent's illness. This indicates that the ill parent and health care providers to a large degree do not speak with the children about mental disorders in parents. The reasons for this are most likely multifaceted, but nevertheless, researchers have postulated that a lack of information may result in an elevated risk of negative developmental outcomes and may run counter to the children's own wishes, needs and rights (7, 36, 38).

The following factors increased the likelihood that the children received information about the parent's condition and treatment/hospitalization: (1) that the child was living with a single parent with a mental illness, (2) that the child was an older child/teenager, (3) that the mother was the parent with a mental illness, (5) that the parent's primary diagnosis was a severe mental illness, and specifically (6) that the parent's disorder was a personality disorder, schizophrenia, or other psychotic disorder.

If the child lived alone with a single parent with a mental illness, the odds that the child had received information were significantly higher. Children who lived with a single parent at all times had almost three times the odds of receiving information about the parent's illness, as compared to children from two-parent homes and children who primarily lived with the other, healthy parent. This may imply that the parent with a mental illness or other adults realized that the child's opportunity to understand and handle their situation would depend on having information about the health status of their primary caregiver.

There were large age differences related to whether the children received information, and the odds that the children received information were higher for each year they grew older. The oldest children were far more likely to receive information about the treatment/hospitalization and about the parent's condition compared to the youngest children. Intuitively, this can be interpreted as a result of older children understanding more about the parent's illness, and thus to a greater extent taking the initiative to talk about it. However, studies have shown that few COPMI seek help from health care providers or their social network (34, 36), which may explain the fact that many of the older children in fact had not received information. It may also be the case that adults feel safer talking to children about a parent's illness when the children are more mature and have a more developed language. Although it could be expected that children under 2 years had generally not received information, the results showed that it was only from age 10 that more than half of the children had received information about the parent's illness. However, school-aged children have both the verbal and cognitive ability to understand phenomena, such as mental illness and substance abuse disorders and they will have perceived that their parents have problems of this type. Therefore, the finding that many children did not receive information about the parent's illness at all could be interpreted as a reflection of that parents and health care providers do not know how to talk to the youngest children about mental illness.(3) For the youngest children this may be due to a culture where adults (both parents and health care providers) have a desire to protect the younger children from “bad things.” However, younger children will also be affected by the parent's psychopathology, sometimes even more than older children since they are more depending of predictable daily routines and sensitive care. Therefore, they will also need information adjusted to their age and maturity to help them understand the parent's symptoms, as well as how these affects parenting and the daily routines in the family. In circumstances when someone other than the parents need to take care of the child, an explanation for this should also be offered to the child.

Children of mentally ill mothers were more likely to receive information than the children of mentally ill fathers. This may be partly explained by children often having closer emotional ties to mothers than to fathers, that mother-daughter relationships often is characterized by emotional closeness and increased communication (60), as well as the fact that more of the children lived with their mother than their fathers.

More serious disorders were associated with a greater probability that the child had received information. This may be because these disorders to a greater extent lead to functional impairments in parents, which increases the negative impact on the child and thus the necessity of the child receiving information about the condition.

Children of parents with personality disorders, schizophrenia, schizotypal disorder and other paranoid disorders, also had higher odds of receiving information. A possible explanation for this may be related to the parents having such significant functional impairments and behaving so differently that it would be difficult to keep information about this from the children. For example, psychoses and delusions may present in ways that are very frightening for children and thus necessitate an explanation earlier than many other types of disorders. Again, substance abuse disorders differed from other diagnosis as it led to lower odds that the child received information. Substance abuse disorders are subject to great social stigma (61), and this may be part of the reason that parents are strongly motivated to hide their disorder from their children. It may also be the case that these parents are more worried about losing custody of their children, compared to parents with other mental disorders, and as such openness would seem counterproductive. The stigma of substance abuse disorders may also explain why personnel in mental health care services do not inform the children. Unfortunately, there are few interventions available for children whose parents have severe mental illnesses in Norwegian municipalities. Such interventions should be adopted from other countries, translated, and adapted to the Norwegian context, or developed locally. All new practices should be evaluated to gain knowledge about effects and feasibility.

### Limitations

The most important limitation in this study is that the information is based on parents' reports. Other sources of information, such as interviews with the children themselves, as well as supplementary information from teachers, nurses and other relatives, would have given a more accurate, detailed, and comprehensive picture of the children's situation. However, gathering information in this way was not within the scope of the quality assurance framework in this study.

Another limitation is associated with the analyses. To be able to perform a binary logistic regression where two information to the children variables were included, the response “partially” was coded as “yes.” These analyses must therefore include the caveat that “partially” could mean anything from almost no information to quite a bit information. Moreover, the extent to which the categorization of diagnoses according to ICD chapters reflect patients' symptom pressures and daily functioning is uncertain. Additionally, many of the diagnostic categories did not have a sufficiently large selection to be included in the analyses, and were therefore excluded.

The categorization of diagnoses by severity was done in accordance with Kessler et al. (22) categorization. They included separate functional goals in their study, which was not included in this study. The severity categorization was therefore only based on the diagnoses, and no controls was made for the fact that psychiatric disorders and their outcomes represent a spectrum of outcomes rather than exact functional measurements. There may therefore be limited correspondence between the assumed and actual degree of severity.

## Conclusion and Implications for Future Clinical Practice and Research

The need for new measures seem to be especially significant for COPMI aged 0–5, as this group seems to be the most invisible, and most vulnerable group, while at the same time having few services offered. The finding that this group also receive the least information about their parent's psychopathology, legitimize significant concern. Norwegian law requires health care providers to attend to the children's need for follow-up, but also to ensure that the children receive the necessary information. The youngest group of COPMI is an important target group for early interventions, according to both national and international manifestos, and measures should therefore be developed to support these children and their parents.

The finding that only half of the children receive information about the parent's psychopathology clearly demonstrates that mental health services for adults must be enabled to comply with current legislation. Close to a third of the children live alone with a mentally ill parent, and thus may not have daily access to an adult who can compensate for the functional impairment the parent suffers in their everyday life at home. These children will need significant others to support their development. Enhancing inter-service collaboration seems crucial to reduce the risk that COPMI will develop social problems and mental health disorders themselves.

Based on the findings in this study, there seems to be a strong cause for concern about the continued transmission of psychiatric disorders from one generation to the next. Parents need support and help to inform their children about their mental health problems, and this is especially important for parents of young children. The findings in this study demonstrate how identifying COPMI and their living arrangements, can inform mental health workers about which type of support and interventions their patients need the most.

## Ethics Statement

The data drawn from electronic patient journals in this study is information which health personnel is required by law to obtain. The Data Protection Officer at the University Hospital of Northern Norway approved the quality evaluation project.

## Author Contributions

All authors agree to be accountable for the content of the work. CR and CL designed the project. CR collected the data. Analyses were conducted by KR, YS, JF, and CR. CR drafted the article. All authors participated in the writing of the manuscript.

### Conflict of Interest Statement

The authors declare that the research was conducted in the absence of any commercial or financial relationships that could be construed as a potential conflict of interest.

## Footnotes

^1^Warren E. New Survey Shows That Mental Health is a “Nonversation” for Parents (2015). Available online at: http://www.time-to-change.org.uk/news/new-survey-shows-mental-health-%E2%80%98nonversation%E2%80%99-parents-0

^2^Durkan M, Field F, Lamb N, Loughton T. (2016). Available online at: https://www.1001criticaldays.co.uk/sites/default/files/1001%20days_oct16_1st.pdf
